# Unravelling Bile Viromes of Free-Range Laying Chickens Clinically Diagnosed with Spotty Liver Disease: Emergence of Many Novel Chaphamaparvoviruses into Multiple Lineages

**DOI:** 10.3390/v14112543

**Published:** 2022-11-17

**Authors:** Subir Sarker, Saranika Talukder, Arif Anwar, Thi Thu Hao Van, Steve Petrovski

**Affiliations:** 1 Department of Microbiology, Anatomy, Physiology and Pharmacology, School of Agriculture, Biomedicine and Environment, La Trobe University, Melbourne, VIC 3086, Australia; 2 Faculty of Veterinary and Agricultural Sciences, School of Agriculture and Food, The University of Melbourne, Melbourne, VIC 3010, Australia; 3 Scolexia Pty Ltd., Moonee Ponds, VIC 3039, Australia; 4 School of Science, Bundoora West Campus, RMIT University, Bundoora, VIC 3083, Australia

**Keywords:** spotty liver disease, next-generation sequencing, parvovirus, chaphamaparvovirus, phylogenetics, evolution

## Abstract

Spotty liver disease (SLD) causes substantial egg production losses and chicken mortality; therefore, it is a disease that concerns Australian egg farmers. Over the last few decades, much research has been conducted to determine the etiologic agents of SLD and to develop potential therapeutics; however, SLD still remains a major issue for the chicken industries globally and remained without the elucidation of potentially multiple pathogens involved. To help fill this gap, this study was aimed at understanding the viral diversity of bile samples from which the SLD-causing bacterium, *Campylobacter hepaticus*, has been isolated and characterised. The collected samples were processed and sequenced using high-throughput next-generation sequencing. Remarkably, this study found 15 galliform chaphamaparvoviruses (GaChPVs), of which 14 are novel under the genus *Chaphamaparvovirus*. Among them, nine were complete genomes that showed between 41.7% and 78.3% genome-wide pairwise similarities to one another. Subsequent phylogenetic analysis using the NS1 gene exhibited a multiple incursion of chaphamaparvovirus lineages, including a novel lineage of unknown ancestral history in free-range laying chickens in Australia. This is the first evidence of circulating many parvoviruses in chickens in Australia, which has increased our knowledge of the pathogen diversity that may have an association with SLD in chickens.

## 1. Introduction

The chicken egg industry in Australia is one of the biggest industries supplying food for human consumption and makes a significant contribution to the Australian economy. One of the major issues the industry faces is tackling infectious diseases that can be spread directly or indirectly from one living organism to another. These include bacteria (e.g., *Salmonella*, *Campylobacter*, *Mycoplasma*, *Escherichia coli*, *Avibacterium paragallinarum*, *Ornithobacterium*, *Gallibacterium*, spirochaetosis) and viruses (e.g., infectious bronchitis virus, egg drop syndrome, swollen head syndrome, avian encephalomyelitis, influenza, Newcastle disease, laryngotracheitis) [1,2,3,4]. The emergence of spotty liver disease (SLD), caused by *Campylobacter hepaticus*, has been recognised by the poultry industry in Australia (especially the free-range laying poultry sector) as ‘one of the most important disease challenges for the Australian egg industry’ [2]. In addition to this pathogen being a significant problem in Australia, it is also a prevalent disease in the United Kingdom, the United States, New Zealand, and Jordan [3,4], and is considered a global poultry issue. SLD can cause an acute reduction in egg production—up to 25%—and mortality can reach up to 30% in affected flocks. As such, the egg farmers of Australia nominated SLD as a ‘priority concern’ [2,5].

The aetiology of SLD was determined in 2015 when a novel *Campylobacter* was isolated from infected birds in England [6]. The following year, the same species was independently isolated and characterised in Australia from the bile samples of SLD-affected chickens [7]. It has been shown that isolated *C. hepaticus* can induce SLD in experimentally inoculated egg-laying chickens [8]. Despite numerous research efforts, there is still no commercially available vaccine or effective method to reduce or eliminate SLD in chickens.

Metagenomics, or metatranscriptomics, is a relatively new technique that enables the detection and characterisation of entire viromes in animals [9,10,11] rather than one single species of virus in isolation. Before metagenomics, there was limited understanding of the viromes present in animal and human hosts. With the advent of metagenomics, information on eukaryotic and prokaryotic viruses, and even on viruses that infect other viruses, has increased [12,13,14,15,16,17]. However, there has been no focus given to investigate the potential presence of viral pathogens in chickens that may or may not be associated with SLD.

To help fill this gap, we performed a virome study on bile collected from free-range chickens showing SLD. Moreover, all the chaphamaparvoviruses that were detected for the first time using high-throughput next-generation sequencing were further analysed to reveal their diversity and evolutionary history. Members of family *Parvoviridae* are small, non-enveloped, linear, single-stranded DNA genomes of 4–6 kb [18]. Viruses in two subfamilies, the *Parvovirinae* and *Densovirinae*, are distinguished primarily by their respective ability to infect vertebrates (including humans) versus invertebrates [18]. Being genetically limited, most parvoviruses require actively dividing host cells and are host and/or tissue specific. The genus *Chaphamaparvovirus* has been detected in the faecal materials of chickens, turkeys, rats, pigs, and Eidolon helvums, and various tissue samples including serums, rectal swabs, nasal swabs, and lung lavages sourced from pigs [19,20,21,22].

## 2. Materials and Methods

### 2.1. Sampling and Ethical Approval

**Figure 1:**
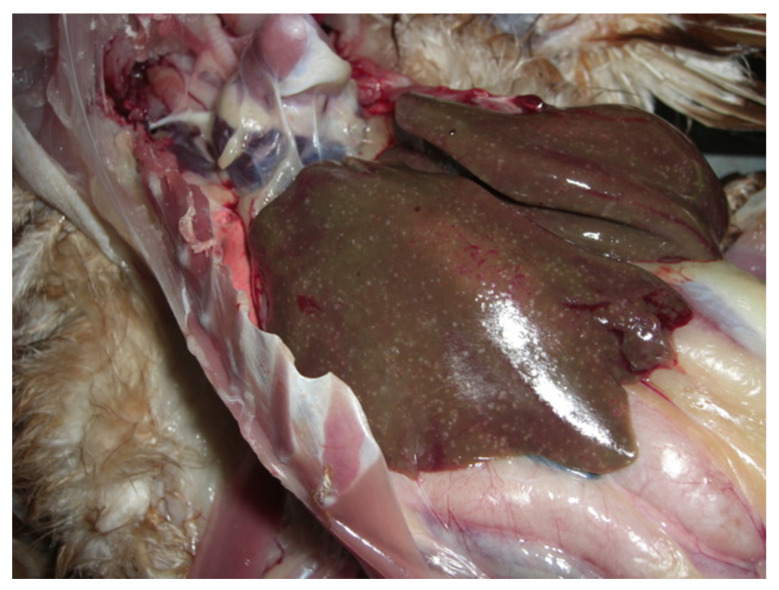
Illustrates liver with necrotic lesions characteristic of spotty liver disease (SLD) in chickens. (Photo courtesy, Dr Arif Anwar, Scolexia Pty Ltd., Moonee Ponds, VIC, Australia).

In 2021, six bile samples were collected from dead chickens pathologically diagnosed with SLD (Figure 1) at a free-range chicken farm in Seymour, Victoria. Samples were collected aseptically (using sterile syringes and needles) directly from the gallbladder, transported in sterile Eppendorf tubes at ambient temperature, and stored at −20 °C temperature until further processing. The reason for choosing bile samples for this study was that the SLD-causing bacterium *C. hepaticus* has been isolated and characterised mainly from the bile of chickens [2,23]. The chickens were necropsied by a registered veterinarian for routine diagnostic purposes, and bile samples were collected using aseptic techniques. The Animal Ethics Committee at La Trobe University was informed that findings from the diagnostic material were to be used in a publication, and a formal waiver of ethics approval was granted.

### 2.2. Virus Enrichment and Virus Nucleic Acid Extraction

Virus particle enrichment was performed under the stated methods after the elimination of impurities (e.g., host cells, bacteria, and free nucleic acids) from bile samples [11,24] with minor variations. Briefly, the bile samples were aseptically resuspended and vigorously homogenised in sterile phosphate-buffered saline (PBS) (1:10) and centrifuged at 2500× *g* for 90 min at 4 °C. The supernatant was filtered using a 0.80 µm syringe filter, and the filtrate was processed downstream. The samples were then ultracentrifuged at 178,000× *g* and 30 psi for one hour at 4 °C using a Hitachi Ultracentrifuge CP100NX (Hitachi Koki Co., Ltd., Tokyo, Japan). The supernatant was discarded, and the pellet was suspended in 130 µL of sterile PBS. The filtrates were then nuclease-treated using 2 µL of benzonase nuclease (25–29 U/µL, purity > 90, Millipore; Merck KGaA, Darmstadt, Germany) and 1 µL of micrococcal nuclease (2,000,000 gel units/mL; New England Biolabs, Ipswich, MA, USA) and incubated at 37 °C for two hours. The nuclease reaction was stopped by adding 3 µL of 500 mM ethylenediaminetetraacetic acid. The viral nucleic acids were extracted using a QIAamp Viral RNA Mini Kit (Qiagen, Valencia, CA, USA) without carrier RNA, which allowed the simultaneous extraction of viral DNA and RNA. The quantity and quality of the isolated nucleic acids were determined using a Nanodrop and an Agilent Tape Station at the Genomic Platform, La Trobe University.

### 2.3. Next-Generation Sequencing

Before library construction, pooled extracted nucleic acids were subjected to cDNA synthesis, and amplification was carried out using the Whole Transcriptome Amplification Kit (WTA2, Sigma-Aldrich, Darmstadt, Germany) as per manufacturer instructions. Amplified polymerase chain reaction (PCR) products were then purified using the Wizard^®^ SV Gel and PCR Clean-Up kit (Promega, Madison, WI, USA). The quantity and quality of the purified products were checked using a Qubit dsDNA high-sensitivity assay kit with Qubit Fluorometer v4.0 (Thermo Fisher Scientific, Waltham, MA, USA). The library construction was performed as a pool that contained six samples using the Illumina DNA Prep (Illumina, San Diego, CA, USA) as per kit instructions, starting with 250 ng of DNA as measured by a Qubit Fluorometer v4.0 (Thermo Fisher Scientific, Waltham, MA, USA). The quality and quantity of the prepared library were assessed by the Australian Genome Research Facility, Melbourne, Australia. According to the manufacturer’s instructions, cluster generation and sequencing of the pooled library were performed with 150 bp paired-end reads on the Illumina^®^ NovaSeq chemistry.

### 2.4. Bioinformatic Analyses

The resulting 47.7 million raw sequencing reads were analysed as per the established pipeline [25,26,27,28] using Geneious Prime^®^ (version 2022.1.1, Biomatters, Auckland, New Zealand) and CLC Genomics Workbench (version 9.0.1, CLC bio, a QIAGEN Company, Prismet, Aarhus C, Denmark). Briefly, preliminary quality evaluation for all raw reads was generated and preprocessed to remove ambiguous base calls and poor-quality reads (trim using quality score limit of 0.05 and trim ambiguous nucleotides up to 15 using CLC Genomics Workbench) and trimmed to remove the Illumina adapter sequences. Trimmed sequence reads were mapped against the chicken genome *Gallus* (GenBank Accession No. NC_006088.5) to remove likely host DNA contamination. In addition, reads were further mapped to *Escherichia coli* bacterial genomic sequence (GenBank Accession No. U00096) to remove possible bacterial contamination. A total of 45.5 million cleaned and unmapped reads were used as input data for de novo assembly using a SPAdes assembler (version 3.10.1) [29] under the ‘careful’ parameter in the LIMS-HPC system (a high-performance computer specialised for genomics research at La Trobe University, Bundoora, VIC, Australia). The resulting contigs were compared against the nonredundant nucleotide and protein databases on GenBank using BLASTN and BLASTX [30], respectively, with an E-value threshold of 1 × 10^−5^ to remove potential false positives. Contigs that had significant BLAST hits with bacteria, eukaryotes, or fungi were filtered out to remove nonviral reads. Virus contigs of interest greater than 300 nucleotides (nt) were imported into Geneious Prime^®^ (version 2022.1.1, Biomatters, Auckland, New Zealand) for further functional analysis. The detected parvoviruses were annotated using Geneious Prime^®^ (version 2022.1.1, Biomatters, Auckland, New Zealand), where galliform chaphamaparvovirus 3 (GaChPV-3, GenBank Accession No. MW306779) was used as a reference guideline.

### 2.5. Comparative Genomics and Phylogenetic Analyses

Genomic features of the newly sequenced parvoviral genomes were visualised using Geneious Prime^®^ (version 2022.1.1, Biomatters, Auckland, New Zealand). Sequence similarity percentages between representative viruses were determined using tools available in Geneious Prime^®^ (version 2022.1.1, Biomatters, Auckland, New Zealand). For phylogenetic analysis, demonstrative parvoviral gene sequences were downloaded from GenBank, and trees were constructed using CLC Genomics Workbench (version 9.0.1, CLC bio, a QIAGEN Company, Prismet, Aarhus C, Denmark). The amino acid sequences of protein-coding genes of 75 nonstructural 1 (NS1) proteins were aligned using the MAFTT L-INS-I algorithm (version 7.388) [31] implemented in Geneious Prime^®^ (version 2022.1.1, Biomatters, Auckland, New Zealand). Phylogenetic analysis was performed using the WAG substitution model, with 1000 bootstrap replicates in CLC Genomics Workbench (version 9.0.1, CLC bio, a QIAGEN Company, Prismet, Aarhus C, Denmark).

## 3. Results

### 3.1. Genomic Characteristic and Diversity of Sequenced GaChPV

A total of nine complete and six partial genomes of GaChPVs were sequenced and assembled from the bile of free-range laying chickens showing gross pathology consistent with SLD. The length of the GaChPV genomes sequenced in this study ranged from 4367 bp to 4032 bp (Supplementary Table S1). Except for GaChPV-2 (GenBank Accession No. OM920509), a genome sequenced in this study that shows the highest similarity with a previously isolated GaChPV-2 (87.39% and 88.26% for the genomic level and protein sequence of NS1 gene, respectively), all other genomes of GaChPV were highly divergent (Table 1 and Table 2). According to the recent nomenclature of considering species of parvovirus (i.e., same species if their NS1 proteins share more than 85% amino acid sequence identity) [18], our study detected 14 GaChPVs demonstrating less than 80% similarity at both genomic and individual NS1 protein levels (Table 1 and Table 2); therefore, all 14 GaChPVs found are to be considered as novel parvoviruses under the genus *Chaphamaparvovirus*, family *Parvoviridae*, and subfamily *Hamaparvovirinae*.

**Figure 2:**
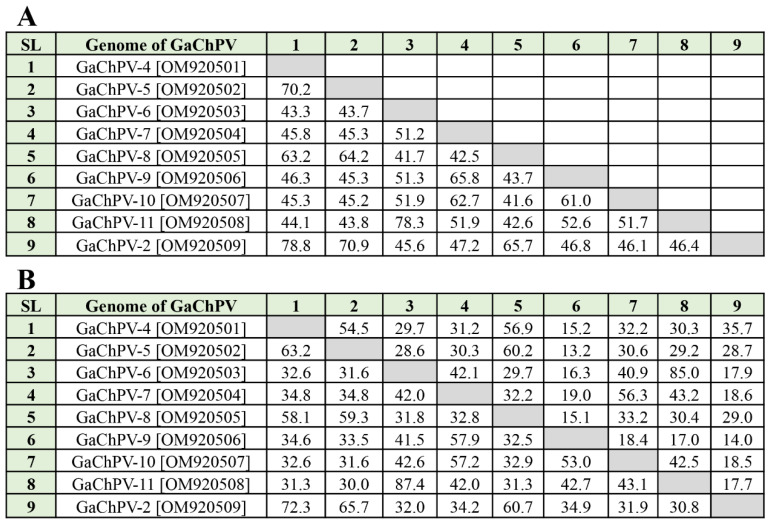
Comparison of galliform chaphamaparvovirus (GaChPV) genome sequences in this study. (**A**) Pairwise similarities percentage among GaChPV genome sequences in this study. (**B**) Pairwise comparison among GaChPV complete coding sequences of major genes. Upper comparison panel indicates the similarities percentage between two sequences of capsid gene (VP1), and lower comparison panel indicates the similarities percentage between two sequences of nonstructural protein 1 (NS1). SL denotes serial number.

Nine complete genomes of GaChPV sequenced in this study showed between 41.7% and 78.3% genome-wide pairwise similarity to one another, and 64.1% to 87.4% similarity to the other parvovirus genomes available in GenBank that have been isolated globally (Table 1 and Figure 2). The highest numbers of chaphamaparvoviruses sequenced in this study (GaChPV-4, GaChPV-5, GaChPV-6, GaChPV-7, GaChPV-9, GaChPV-10, GaChPV-2, GaChPV-12, GaChPV-13, GaChPV-14, GaChPV-16, and GaChPV-17) showed topmost similarity with other chaphamaparvoviruses detected in other galliform species (e.g., chicken, turkey, and peafowl) (Table 1). Importantly, three chaphamaparvoviruses sequenced in this study (GaChPV-8, GaChPV-11, and GaChPV-15) exhibited the highest similarities with the chaphamaparvoviruses isolated from chestnut teal ducks (*Anas castanea*) in Australia [32], tundra swans (*Cygnus columbianus*) in China and black swans (*Cygnus atratus*) in China–73.73%, 76.22%, and 71.59%, respectively.

### 3.2. Comparative Analyses of Coding Genes of GaChPV

**Figure 3:**
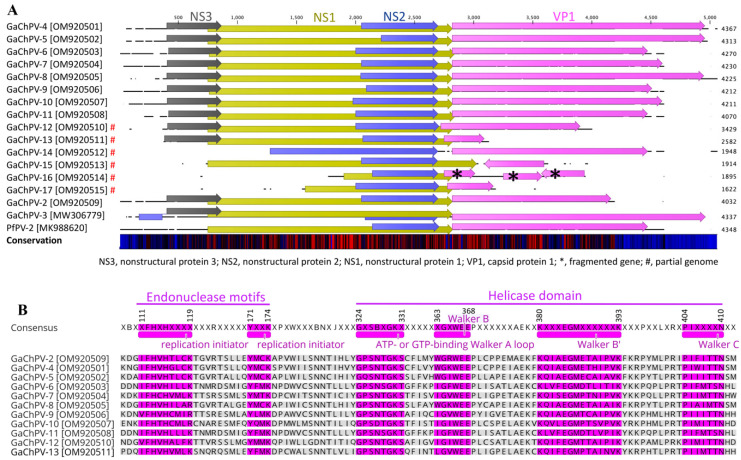
A schematic illustration of the selected avian chaphamaparvoviruses. (**A**) A schematic map of the galliform chaphamaparvoviruses (GaChPVs) sequenced in this study (GenBank Accession No. OM920501–OM920515) compared to GaChPV-3 (GenBank Accession No. MW306779) and the peafowl parvovirus 2 (PfPV-2, GenBank Accession No. MK988620), using the CLC Genomic Workbench (version 9.0.1). The arrows symbolise chaphamaparvovirus genes and open-reading frames (ORFs) predicted to code for proteins, indicating their transcriptional direction. Each gene or ORF is colour coded. The bottom graph represents the sequence conservation among the aligned selected sequences at a given coordinate at each position in the alignment. The colour gradient reflects the conservation of that position in the alignment. Red presents 100 conserved regions across all viruses, black is 50 conserved regions, and blue is less than 50 conserved regions. * = denotes genes fragmented; # = denotes partial genomes. (**B**) Alignment of conserved domains of nonstructural protein 1 (NS1) from 11 GaChPVs sequenced in this study.

At the genomic level, GaChPV genomes (GaChPV-2 GaChPV-4 to GaChPV-11) contained four genes with the same relative order and orientation (Figure 3). Furthermore, there were several occurrences of gene truncation and elongation observed among GaChPVs detected in this study (Figure 3 and Table 2).

The NS1 genes of GaChPVs detected in this study demonstrate significantly low sequence similarities compared to other parvovirus isolates (Figure 3A and Table 2). At the amino acid level, GaChPV NS1 genes exhibit 45.29% to 88.26% identities compared to other parvoviruses (Table 2). The NS1 genes of GaChPV-2 and GaChPV-11 show the highest and lowest amino acid similarities to the previously identified GaChPV-2 (protein similarity 88.26.%, GenBank Accession No. AXL64657.1) and peafowl parvovirus 2 (protein similarity 45.29.%, GenBank Accession No. QGJ83204.1). Except for the NS1 gene of GaChPV-15, which shows the highest amino acid similarity to the previously identified duck-associated chaphamaparvovirus 2 (protein similarity 54.73.%, GenBank Accession No. QRK03694.1), all other NS1 genes of GaChPVs detected in this study show the highest amino acid similarities with chaphamaparvoviruses detected in other galliform species (e.g., chicken, turkey, and peafowl) (Table 2).

Like other parvoviruses, the lengths of complete NS1 genes of 11 GaChPVs detected in this study range from 662 to 673 amino acids, and they encode helicases, including the conserved ATP- or GTP-binding Walker A loop (GPxNTGKT/S; _324_**G**xSBx**GKT/S**_331_), Walker B (xxxWEE; _363_xGx**WEE**_368_), Walker B’ (KQxxEGxxxxxPxK; _380_**K**xxx**EG**Mxxxxxx**K**_393_) and Walker C (PxxxTxN; _404_**P**Ixxxx**N**_410_) as motifs. In addition, the NS1 protein contains two conserved replication initiator (endonuclease) motifs, xxHuHxxxx (xF_112_**H**u**H**_115_xxxx) and YxxK (_171_**Y**xx**K**_174_). (Conserved amino acids are indicated in bold letters, and u indicates a hydrophobic residue; residue numbers correspond to position in consensus sequence) (Figure 3B).

All the GaChPV genomes detected in this study were predicted to contain an open-reading frame (ORF), which was shown to be homologous to the NS2 gene of other parvoviruses (Figure 3A and Table 2). At the amino acid level, the complete NS2 gene of GaChPV exhibited 53.63% to 81.14% identities compared to other parvoviruses (Table 2). Except for the NS2 protein of GaChPV-5, GaChPV-8, GaChPV-11, and GaChPV-15, which show the highest amino acid similarities (from 52.75% to 72.67%) to the previously identified wood duck chaphamaparvovirus (WDChPV), chestnut teal chaphamaparvovirus 1 (CTChPV-1), and ara ararauna parvoviridae sp. (AAPV), all other NS2 genes of GaChPVs detected in this study show the highest amino acid similarities with chaphamaparvoviruses detected in other galliform species (e.g., chicken and peafowl) (Table 2). The NS3 genes of GaChPVs detected in this study show similarities with other parvoviruses previously isolated in a range from 42.86% to 74.83% (Table 2). Importantly, only 4 out of 11 GaChPVs that encoded the NS3 gene (GaChPV-2, GaChPV-4, GaChPV-5, and GaChPV-8) demonstrated significant similarities (>60%) with GaChPVs detected in other galliform species.

The major 3′ ORF is the structural *Parvoviridae* capsid protein VP1. Except for the VP1 gene of GaChPV-2, which shows the highest similarity with a previously isolated GaChPV-2 from chickens, all other GaChPV VP1 genes exhibited somewhat low amino acid similarities (44.04% to 70.02%) to the VP1 of other parvoviruses. Except for the VP1 gene of GaChPV-5, GaChPV-8, GaChPV-9, GaChPV-13, GaChPV-14, and GaChPV-15, which show the highest amino acid similarities to the previously identified CTChPV-1, DAChPV-1, pavo cristatus parvoviridae sp. (PCPV), Parvoviridae sp. (RcPV), and WDChPV (59.33%, 60.34%, 65.00%, 63.95%, 48.04%, and 51.02%, respectively), all other VP1 genes of GaChPVs detected in this study show the highest amino acid similarities with chaphamaparvoviruses detected in other galliform species (e.g., chicken, turkey, and peafowl) (Table 2).

### 3.3. Emergence of GaChPV into Multiple Lineages

**Figure 4:**
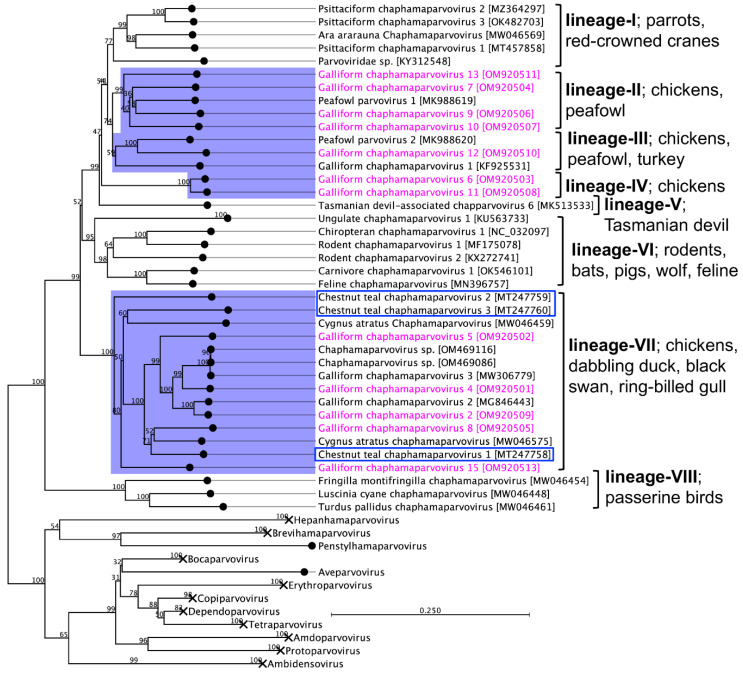
Maximum likelihood phylogenetic tree shows the possible evolutionary relationship of novel galliform chaphamaparvoviruses (GaChPVs) detected in this study with other selected parvoviruses. The numbers on the left show bootstrap values as percentages, and the labels at the branch tips refer to the original parvoviruses’ species names (followed by the GenBank accession numbers in parentheses). The clade correspondence to the chaphamaparvoviruses sequenced in this study has a purple background, and the GaChPVs sequenced in this study are shown in pink. All other clades relevant to other genera are collapsed (details tree under Supplementary Figure S1). Blue box indicates chaphamaparvoviruses sequenced previously from Australian birds.

Phylogenetic analysis based on complete NS1 protein-coding sequences of 12 GaChPVs discovered in this study and other representative parvoviruses retrieved from the GenBank clearly supported that there was a multiple incursion of chaphamaparvovirus lineages in the free-range chicken farm in Australia chosen for this study (Figure 4). Although the amino acid sequence similarities among NS1 genes of 12 GaChPVs were within a range of 45.29% to 88.26%, the GaChPVs sequenced in this study were clustered into four distinctive lineages in the resulting maximum likelihood (ML) tree. Lineage II, consisting of four GaChPVs isolated in this study (GaChPV-7, GaChPV-9, GaChPV-10, and GaChPV-13 with GenBank Accession Nos. OM920504, OM920506, OM920507, and OM920511, respectively) demonstrated a strong clade support (99%) with a chaphamaparvovirus—peafowl parvovirus 1—and shared from 51.1% to 58.7% amino acid identities among them. GaChPV-12, an isolate sequenced in this study (GenBank Accession No. OM920510) showed the strongest clade support (100%) with two other chaphamaparvoviruses: peafowl parvovirus 2 and galliform chaphamaparvovirus 1, which was sequenced from Indian peafowl (*Pavo cristatus*) and domestic turkeys (*Meleagris gallopavo*) in China [33] and Hungary [19] (Lineage III), indicating that the GaChPVs found in this study may have originated from a different ancestor. Strikingly, two GaChPVs sequenced in this study (GaChPV-6 and GaChPV-11; GenBank Accession Nos. OM920503 and OM920508, respectively) emerged as a novel lineage (Lineage IV) with a strong bootstrap support (100%), indicating that the chosen chicken farm may be circulating unique chaphamaparvoviruses that have never been reported. Lineage VII is dominated by the chaphamaparvoviruses isolated from chickens in Switzerland and Australia, dabbling ducks (*Anas castanea*) in Australia [32], and black swans (*Cygnus atratus*) in China and the GaChPV-3, which has been isolated from ring-billed gulls (*Larus delawarensis*) in Canada [34]. Most strikingly, it is evident that chaphamaparvoviruses in Australia may have first evolved in a bird species–dabbling duck (*Anas castanea*) [32]–that was present before the Galliformes (mainly chickens) and Charadriiformes (mainly ring-billed gulls, *Larus delawarensis*) (Lineage VII, Figure 4). Based on the ML tree (Lineage VII), it appears that some chaphamaparvoviruse*s* detected in this study potentially originated in ducks; then, host-switching events resulted in infections in galliform birds and several other bird species.

## 4. Discussion

This study presents evidence of many highly divergent novel chaphamaparvoviruses in the bile of free-range laying chickens showing gross pathology consistent with SLD. Strikingly, this study reports for the first time 14 novel GaChPVs circulating in laying chickens that show from 64.1% to 87.4% similarities to other parvoviruses isolated globally. All novel genomes of GaChPV sequence derived from free-range laying chickens contain all the major structural and functional genes, and NS1 genes exhibit from 45.29% to 88.26% amino acid identities compared to other parvoviruses. Except for the NS1 gene of GaChPV-2, all other NS1 genes of GaChPVs sequenced in this study are significantly divergent and clearly represent separate species, as demonstrated by the low degree of amino acid identities (between 45.29% and 77.63%) to the closest parvovirus genes. Following the recent nomenclature of considering species of parvovirus [18], we propose the 14 GaChPVs found to be novel parvoviruses under the genus *Chaphamaparvovirus*, family *Parvoviridae*, and subfamily *Hamaparvovirinae*.

Although this is an interesting finding and opens a lot of opportunities for further investigation, some aspects of this case are difficult to explain fully without conducting ethically debatable virus-transmission experiments. The direct or indirect effect of these parvoviruses on the livers of laying chickens is unknown. However, a recent finding in another Galliformes species–pheasants (*Phasianus colchicus*)–shows several outbreaks of hepatitis with high mortality, which were caused by a novel chaphamaparvovirus. The aetiology of hepatitis outbreaks in pheasants was confirmed by pathology, sequencing, and in situ hybridisation [35]; pathologically, extensive areas of severe diffuse degeneration and hepatic necrosis with sparse infiltration of mononuclear inflammatory cells and heterophils were observed in liver tissue, with degenerative hepatocytes presenting large amphophilic to acidophilic intranuclear inclusion bodies. Additionally, parvovirus strains from the *Anseriform dependoparvovirus 1* species within the *Dependoparvovirus* genus are also capable of causing acute disease in young geese and ducks, with characteristic lesions (including hepatitis) and the presence of similar intranuclear inclusion bodies [36]. In contrast, the etiological agent of the SLD-causing bacterium, *C. hepaticus*, is isolated and characterised from the bile of laying chickens, and the organism produces histological changes in liver tissue [2,5] very similar to hepatitis in pheasants (*Phasianus colchicus*) [35]. However, authors [2] reported that infection with *C. hepaticus* alone may not be sufficient to induce disease; some other predisposing factors may also be required [2]; this claim is supported by various previous research efforts [5]. For example, due to the absence of bacteria in necrotic hepatic foci, it has been suggested that a bacterial toxin, such as a member of the cytolethal distending toxin (CDT) group commonly found in *C. jejuni*, could be associated with liver pathology [37]. In contrast, a study by [38] reported that CDT genes were not detected in their studies of *C. hepaticus*. Though the presence of toxin-induced pathology cannot be discounted, the influence of such entities in *C. hepaticus* pathology remains inconclusive. It is likely possible that there are other pathogens, including viruses that might contribute to the development of liver pathology, which require further investigation. The pathogenic effects of parvoviruses detected in this study on the liver are largely unknown; however, considering that several outbreaks of hepatitis in pheasants [35], geese, and ducks [36] were caused by parvoviruses, the long-term consequences in susceptible populations and their pathology remain to be determined.

Our phylogenetic analysis provides strong evidence of multiple circulating chaphamaparvovirus lineages in the free-range chicken farm in Australia (Figure 4) and supports that the newly sequenced GaChPVs are representative species of the genus *Chaphamaparvovirus*. The detected GaChPVs are divergent from the viruses belonging to other genera of the subfamily. Thus, our results suggest that there are multiple lineages, including a unique lineage of chaphamaparvoviruses that has emerged during the evolution of parvoviruses in free-range chickens in Australia. Interestingly, the GaChPVs detected in this study were shown to be highly divergent genetically from chaphamaparvoviruses that infect other galliform species, such as chickens, turkeys, and peafowl [19,33]. In the absence of any parvovirus sequence in Australian chickens, it is difficult to elucidate the origin of the GaChPVs from this case alone. It is likely that these viruses are circulating in free-range and/or captive chickens in Australia but have not been detected yet. In support of our findings, two recent studies on peafowl [33] and pheasants [35], in which the authors discovered novel chaphamaparvoviruses in tissues, showed that case fatalities were linked to parvoviruses; however, many viruses designated as *Chaphamaparvovirus* were identified from faecal samples [19,22,24,39], swabs [20], bile [40], or asymptomatic tissues [41].

Interestingly, CTChPV-2 is basal to many known avian chaphamaparvoviruses, including five GaChPVs detected in this study (Lineage VII, Figure 4), which suggests that all the avian chaphamaparvoviruses clustered in this lineage may have evolved from the ancestral duck species that gave rise to CTChPV-2. The duck species (chestnut teal, *Anas castanea*) from which CTChPV-2 was isolated, is one of the few species of Australian ducks [32] and is found in southwestern and southeastern Australia, Rockhampton, Queensland to Ceduna, South Australia, and is most common in New South Wales, Victoria and Tasmania. However, it is not documented whether free-range chickens intimately share any ecological niche with any known duck species. Free-range chickens are usually reared to roam and forage outdoors for at least eight hours a day, an activity that has increased in the last decades. Such husbandry systems present more biosecurity challenges than conventional poultry farms, implying a higher risk of the introduction and transmission of pathogens. A possible scenario for transmission could be the ingestion of wild duck-contaminated food within their pasture since parvovirus is likely to persist in the environment for a time, facilitating exposure to susceptible animals [42]. It is well known that wild birds harbour a number of bacterial, fungal, viral, and parasitic diseases, which can be transmitted to captive and/or free-range poultry; the potential for diseases to transfer from wildlife to poultry may increase when poultry have access to the outdoors [43].

## 5. Conclusions

We have reported the emergence of many novel chaphamaparvoviruses circulating in free-range laying chickens in Australia. The GaChPV genomes are structurally similar to those of other chaphamaparvoviruses and likely originated from a common ancestor that deviated from its GaChPV-like progenitor. This is the first report of the detection of a parvovirus in chickens in Australia, and the findings of this study have increased our knowledge of pathogen diversity among chickens in Australia. These findings highlight important and unexpected aspects of likely parvovirus disease emergence and host-switching or host–virus coevolution pertinent to other situations when viruses undergo host-switching across relatively deep phylogenetic divides. Additional investigations will be required to better understand relevant host–pathogen dynamics, including routes of transmission and factors leading to infection, associated pathology, and disease prevalence in other organs/tissues.

## Figures and Tables

**Table 1:** Comparative analysis of galliform chaphamaparvovirus (GaChPV) sequenced in this study.

SL	GaChPV (GenBank Accession No.)	Length (nt)	Best BlastN Match (Organism/Query Coverage (%)/E-Value/GenBank Accession No.)	nt Similarity (%)
1	GaChPV-4 (OM920501)	4367	Galliform chaphamaparvovirus 2/97/0.0/MG846442.1	79.90
2	GaChPV-5 (OM920502)	4311	Galliform chaphamaparvovirus 3/63/0.0/MW306779.1	74.90
3	GaChPV-6 (OM920503)	4270	Peafowl parvovirus 2/33/3.00 × 10^−44^/MK988620.1	64.11
4	GaChPV-7 (OM920504)	4230	Peafowl parvovirus 1/29/1.00 × 10^−125^/MK988619.1	73.95
5	GaChPV-8 (OM920505)	4225	Chestnut teal chaphamaparvovirus 1/87/0/MT247758.1	73.73
6	GaChPV-9 (OM920506)	4212	Pavo cristatus parvoviridae sp./30/0/MW046349.1	76.56
7	GaChPV-10 (OM920507)	4211	Peafowl parvovirus 1/36/6.00 × 10^−79^/MK988619.1	71.12
8	GaChPV-11 (OM920508)	4070	Cygnus columbianus Chaphamaparvovirus/19/4.00 × 10^−30^/MW046623.1	76.22
9	GaChPV-2 (OM920509)	4032	Galliform chaphamaparvovirus 2/97/0.0/MG846443.1	87.39
10	GaChPV-12 (OM920510)	3429	Peafowl parvovirus 2/67/2.00 × 10^−127^/MK988620.1	71.15
11	GaChPV-13 (OM920511)	2582	Peafowl parvovirus 2/30/6.00 × 10^−62^/MK988620.1	73.30
12	GaChPV-14 (OM920512)	1948	Parvoviridae sp./19/6.00 × 10^−17^/MT138323.1	67.62
13	GaChPV-15 (OM920513)	1914	Ara ararauna Chaphamaparvovirus/52/4.00 × 10^−52^/MW046364.1	71.59
14	GaChPV-16 (OM920514)	1894	Pavo cristatus parvoviridae sp./77/0.0/MW046349.1	78.66
15	GaChPV-17 (OM920515)	1622	Peafowl parvovirus 2/92/0.0/ MK988620.1	71.29

**Table 2:** Comparative analysis of ORFs of galliform chaphamaparvovirus (GaChV) detected in this study.

NS3					NS2			NS1			VP1		
SL No	Genome (GenBank Accession No)	Gene Coordinate (nt Length)	AA Similarity (%)	Best BLAST Match	Gene Coordinate (nt Length)	AA Similarity (%)	Best BLAST Match	Gene Coordinate (nt Length)	AA Similarity (%)	Best BLAST Match	Gene Coordinate (nt Length)	AA Similarity (%)	Best BLAST Match
1	GaChPV-4 (OM920501)	297–740 (444)	74.83	NS3 (GaChV-3, QRK03700.1)	1917–2525 (609)	81.14	GaChV-3, QRK03699.1	623–2647 (2025)	77.63	GaChV-3, QRK03698.1	2644–4317 (1674)	70.02	GaChV-3, QRK03701.1
2	GaChPV-5 (OM920502)	285–728 (444)	69.39	NS3 (GaChV-3, QRK03700.1)	2067–2519 (453)	72.67	WDChPV, QMI57952.1	611–2629 (2019)	64.45	GaChV-3, QRK03698.1	2626–4290 (1665)	59.33	CTChPV-1, QMI57831.1
3	GaChPV-6 (OM920503)	343–780 (438)	44.76	ORF1 (ChFV, QSH48278.1)	1906–2580 (675)	53.63	PfPV-2, QGJ83205.1	684–2684 (2001)	45.94	PfPV-2, QGJ83204.1	2659–4173 (1515)	44.04	PfPV-2, QGJ83206.1
4	GaChPV-7 (OM920504)	335–778 (444)	51.35	ORF1 (ChFV, QSH48278.1)	1946–2587 (642)	65.71	PfPV-1, QGJ83202.1	670–2673 (2004)	58.15	PfPV-2, QGJ83201.1	2666–4216 (1551)	56.18	PfPV-1, QGJ83203.1
5	GaChPV-8 (OM920505)	81–524 (444)	61.22	NS3 (GaChV-3, QRK03700.1)	1695–2312 (618)	68.91	CTChPV-1, QMI57830.1	407–2434 (2028)	61.63	GaChPV-2, AXL64657.1	2431–4110 (1680)	60.34	DAChPV-1, QRK03681.1
6	GaChPV-9 (OM920506)	335–778 (444)	42.86	HP (PsChPV-1, QZW33714.1)	1991–2590 (600)	68.97	PfPV-1, QGJ83202.1	685–2673 (1989)	59.21	PfPV-1, QGJ83201.1	2666–4135 (1500)	65.00	PCPV, QTE03716.1
7	GaChPV-10 (OM920507)	307–759 (453)	49.65	HP (PsChPV-1, QZW33714.1)	1858–2571 (714)	64.29	PfPV-1, QGJ83202.1	651–2657 (2007)	56.29	PfPV-1, QGJ83201.1	2650–4194 (1545)	53.76	PfPV-2, QGJ83206.1
8	GaChPV-11 (OM920508)	145–582 (438)	45.14	ORF1 (ChFV, QSH48278.1)	1708–2382 (675)	52.75	AAPV, QTE04008.1	474–2486 (2013)	45.29	PfPV-2, QGJ83204.1	2461–3978 (1518)	46.18	PfPV-2, QGJ83206.1
9	GaChPV-2 (OM920509)	346–789 (444)	74.15	NS3 (GaChV-3, QRK03700.1)	1966–2577 (612)	78.86	GaChV-3, QRK03699.1	672–2693 (2022)	88.26	GaChPV-2, AXL64657.1	2690–3997 (1308)	85.20	GaChPV-2, AXL64658.1
10	GaChPV-12 (OM920510)	61–477 (417)	48.53	ORF1 (ChFV, QSH48278.1)	1600–2280 (681)	67.63	PfPV-2, QGJ83205.1	369–2357 (1989)	59.16	PfPV-2, QGJ83204.1	2287–3327 (1041)	61.41	PfPV-2, QGJ83206.1
11	GaChPV-13 (OM920511)	8–403 (396)	54.00	ORF1 (ChFV, QSH48278.1)	1583–2209 (627)	62.43	PfPV-1, QGJ83202.1	292–2292 (2001)	56.59	PfPV-1, QGJ83201.1	2250–2543 (294)	63.95	PCPV, QTE03716.1
12	GaChPV-14 (OM920512)				2–202 (201)	36.92	PfPV-1, QGJ83202.1				281–1795 (1515)	48.04	RcPV, QKE54986.1
13	GaChPV-15 (OM920513)				908–1525 (618)	63.90	CTChPV-1, QMI57830.1	25–1659 (1635)	54.73	DAChPV-2, QRK03694.1	1830–1681 (150)	51.02	WDChPV, QMI57935.1
14	GaChPV-16 (OM920514)				388–936 (549)	72.13	PfPV-1, QGJ83202.1	147–1022 (876)	55.67	PfPV-1, QGJ83201.1	980–1195 (216)	63.33	PfPV-2, QGJ83206.1
											1430–1729 (300)	45.83	PfPV-1, QGJ83203.1
											1882–1721 (162)	56.60	PfPV-1, QGJ83203.1
15	GaChPV-17 (OM920515)				498–1181 (684)	64.16	PfPV-2, QGJ83205.1	74–1258 (1185)	56.93	PfPV-2, QGJ83204.1	1227–1586 (360)	71.17	PfPV-2, QGJ83206.1

Note: NS3, nonstructural protein 3; NS2, nonstructural protein 2; NS1, nonstructural protein 1; VP1, capsid protein; AA, amino acid; nt, nuclotides; %, percentage; HP, hypothetical protein; GaChV-2, Galliform chaphamaparvovirus 2; GaChV-3, Galliform chaphamaparvovirus 3; PsChPV-1, Psittaciform chaphamaparvovirus 1; PfPV-1, Peafowl parvovirus 1; PfPV-2, Peafowl parvovirus 2; ChFV, Chufflevirus sp.; WDChPV, Wood duck chaphamaparvovirus; CTChPV-1, Chestnut teal chaphamaparvovirus 1; AAPV, Ara ararauna parvoviridae sp.; DAChPV-1, Duck-associated chapparvovirus 1; DAChPV-2, Duck-associated chapparvovirus 2; PCPV, Pavo cristatus parvoviridae sp.; RcPV, Parvoviridae sp.

## Data Availability

All sequences analysed have been deposited in NCBI GenBank under Accession Numbers OM920501-OM920515. Raw sequencing data from this study have been deposited in the NCBI Sequence Read Archive (SRA) under Accession Number SRR19134919 (BioProject ID: PRJNA835504, BioSample Accessions: SAMN28104065) (http://www.ncbi.nlm.nih.gov/sra/, (accessed on 20 September 2022)).

## Supplementary Materials

The following supporting information can be downloaded at: https://www.mdpi.com/article/10.3390/v14112543/s1, Figure S1: Maximum likelihood phylogenetic tree shows the possible evolutionary relationship of novel galliform chaphamaparvoviruses (GaChPVs) detected in this study with other selected parvoviruses. Table S1: Sequencing statistics of GaChPVs detected in this study.
