# Supplementary material for: Unravelling Bile Viromes of Free-Range Laying Chickens Clinically Diagnosed with Spotty Liver Disease: Emergence of Many Novel Chaphamaparvoviruses into Multiple Lineages
Source: Viruses. 2022 Nov 17;14(11):2543. doi: 10.3390/v14112543 (PMC9695665; doi:10.3390/v14112543)
Supplement: Supplementary file 1 [file viruses-14-02543-s001.zip › viruses-1963378-supplementary.pdf]

Supplementary File

Unravelling bile virome of free-range laying chickens clinically diagnosed with spotty liver disease: Emergence of many novel chaphamaparvoviruses into multiple lineages

Subir Sarker [1,*], Saranika Talukder [2], Arif Anwar [3], Thi Thu Hao Van [4], Steve Petrovski [1]

[1] Department of Microbiology, Anatomy, Physiology and Pharmacology, School of Agriculture, Biomedicine and Environment, La Trobe University, Melbourne, VIC 3086, Australia.

[2] School of Agriculture and Food, Faculty of Veterinary and Agricultural Sciences, The University of Melbourne, Melbourne, Victoria, Australia

[3] Scolexia Pty Ltd., Moonee Ponds, VIC, Australia

[4] School of Science, RMIT University, Bundoora West Campus, Bundoora, VIC, Australia

*Correspondence: Department of Microbiology, Anatomy, Physiology and Pharmacology, School of Agriculture, Biomedicine and Environment, La Trobe University, Melbourne, VIC 3086, Australia.
Email: s.sarker@latrobe.edu.au

Supplementary Table S1: Sequencing statistics of GaChPVs detected in this study

GaChPV [GenBank accession no.]	GaChPVs length	Total read count	Average coverage
GaChPV-3 [OM920509]	4003	1581	54.86
GaChPV-4 [OM920501]	4367	2085	66.94
GaChPV-5 [OM920502]	4261	466	14.70
GaChPV-6 [OM920503]	4249	15989	441.15
GaChPV-7 [OM920504]	4194	814	26.44
GaChPV-8 [OM920505]	4152	21289	368.78
GaChPV-9 [OM920506]	4202	3004	100.10
GaChPV-10 [OM920507]	4212	7771	261.48
GaChPV-11 [OM920508]	4070	4699	158.85
GaChPV-12 [OM920510]	3255	345	13.07
GaChPV-13 [OM920511]	2493	315	17.16
GaChPV-14 [OM920512]	1948	13854	1013.05
GaChPV-15 [OM920513]	1891	226	16.00
GaChPV-16 [OM920514]	1827	206	14.47
GaChPV-17 [OM920515]	1622	73	6.41

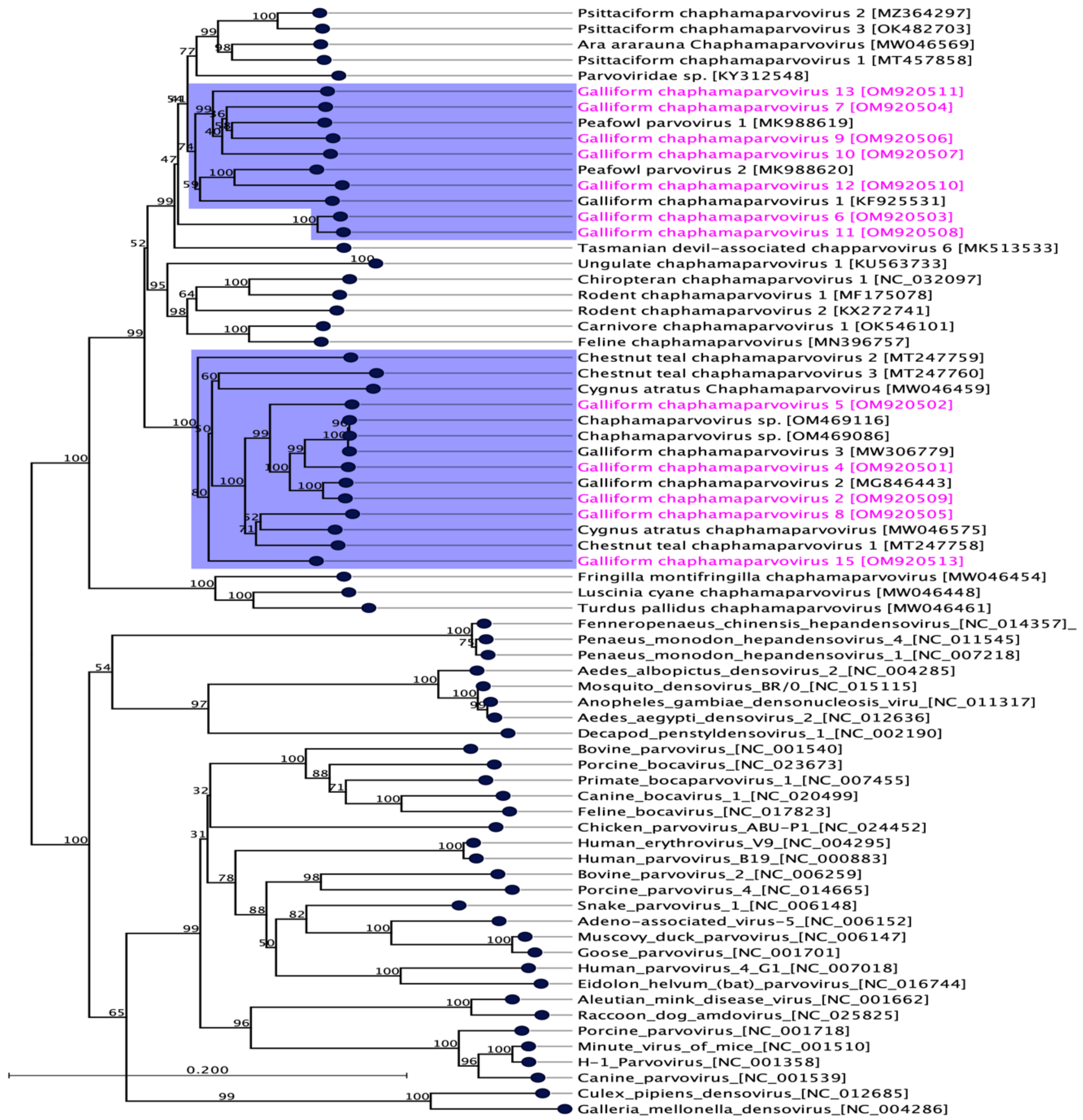

Supplementary Figure S1. Maximum likelihood phylogenetic tree shows the possible evolutionary relationship of novel galliform chaphamaparvoviruses (GaChPVs) detected in this study with other selected parvoviruses. The numbers on the left show bootstrap values as percentages, and the labels at the branch tips refer to the original parvoviruses' species names (followed by the GenBank accession numbers in parentheses). The clade correspondence to the chaphamaparvoviruses sequenced in this study has a purple background, and the GaChPVs sequenced in this study are shown in pink.
